# Increased Body Mass Index and Ventricular Cardiac Magnetic Resonance Characteristics in Adults With Fontan Circulation

**DOI:** 10.1016/j.jacadv.2025.102094

**Published:** 2025-08-25

**Authors:** Ryan D. Byrne, Stephen J. Dolgner, Christopher R. Broda, Tarek Alsaied, Mark Fogel, Timothy C. Slesnick, Rajesh Krishnamurthy, Vivek Muthurangu, Adam L. Dorfman, Christopher Z. Lam, Joshua Robinson, Rahul H. Rathod, Justin D. Weigand, FORCE Investigators, M. Aggarwal, T. Alsaied, A.L. Dorfman, A. Doshi, M.D. Files, M. Fogel, S. Hedge, A. Hoyer, T. Johnson, R. Krishnamurthy, C.Z. Lam, Y. Loke, A.L. Marsden, V. Muthurangu, L.J. Olivieri, M. Quail, F. Raimondi, P. Ramachandran, R.H. Rathod, P. Renella, M.S. Renno, J.D. Robinson, G. Ruchira, A. Shah, T.C. Slesnick, J.H. Soslow, J. Steele, K.W. Stern, B. Thattaliyath, A. Vaikom House, J. Weigand

**Affiliations:** a Adult Congenital Heart Disease Program, Division of Cardiology, Department of Medicine, Vanderbilt University Medical Center, Nashville, Tennessee, USA; b Adult Congenital Heart Program, Department of Pediatrics, Section of Cardiology, Texas Children's Hospital, Baylor College of Medicine, Houston, Texas, USA; c Department of Pediatrics, Section of Cardiology, Baylor College of Medicine/Texas Children's Hospital, Houston, Texas, USA; d The Heart and Vascular Institute, UPMC Children's Hospital of Pittsburgh, University of Pittsburgh School of Medicine, Pittsburgh, Pennsylvania, USA; e Division of Cardiology, Department of Pediatrics, The Children's Hospital of Philadelphia, Perelman School of Medicine, University of Pennsylvania School of Medicine, Philadelphia, Pennsylvania, USA; f Departments of Pediatrics, Emory University School of Medicine, Children's Healthcare of Atlanta, Atlanta, Georgia, USA; g The Department of Radiology, Nationwide Children's Hospital, Columbus, Ohio, USA; h UCL Centre for Cardiovascular Imaging, Institute of Cardiovascular Science, University College London, London, England; i Department of Pediatrics, University of Michigan Medical School, Ann Arbor, Michigan, USA; j Department of Diagnostic Imaging, The Hospital for Sick Children and Department of Medical Imaging, University of Toronto, Toronto, Ontario, Canada; k Department of Pediatrics, Ann & Robert H. Lurie's Children's Hospital of Chicago, Northwestern University Feinberg School of Medicine, Chicago, Illinois, USA; l Department of Pediatrics, Boston Children’s Hospital, Harvard Medical School, Boston, Massachusetts, USA

**Keywords:** adult congenital heart disease, cardiac magnetic resonance imaging, cventricular volume, Fontan, obesity, ventricular mass

## Abstract

**Background:**

In structurally normal hearts, increased body mass index (BMI) is associated with larger ventricular mass and volume, however, this association has yet to be described in adult Fontan patients.

**Objectives:**

This study evaluates the relationship of increased BMI and ventricular characteristics by cardiac magnetic resonance imaging (CMR) in a multi-institutional adult Fontan population.

**Methods:**

We conducted a multicenter, cross-sectional study using the Fontan Outcomes Registry using CMR Examinations. Fontan patients with CMR at ≥16 years of age were included. The primary independent variable was BMI and dependent variables included CMR characteristics, hemodynamics by cardiac catheterization, and clinical outcomes. Multivariable models for CMR characteristics were additionally created. Analyses were stratified by ventricular morphology.

**Results:**

In total, 983 patients were included with 47% morphologic left ventricle (MLV) and 36% morphologic right ventricle. The median age at CMR was 21.9 years. Thirty-nine percent were overweight or obese. Increased BMI was associated with significantly higher end-diastolic volume and ventricular mass (*P* < 0.001 for each). In multivariable analysis, compared to normal weight, end-diastolic volume and ventricular mass were significantly elevated for obese patients (+18.0 mL; 95% CI: 8.3-27.7; *P* < 0.001 and + 19.1 g; 95% CI: 8.7-29.6; *P* < 0.001, respectively). Median ventricular end-diastolic pressure and Fontan pressure were significantly elevated with increased BMI category (*P* = 0.006 and *P* = 0.004, respectively). Stratified by ventricular morphology, similar findings were observed in MLV but not morphologic right ventricle.

**Conclusions:**

Ventricular adaptations in volume and mass incurred by exposure to Fontan circulation are compounded by increased BMI. Obesity may disproportionately affect ventricular volume, mass, and hemodynamics in MLV.

While typically growth restricted in childhood, the prevalence of Fontan patients who are overweight and obese increases in adulthood.^1^^,^^2^ In structurally normal hearts, obesity is associated with increased systemic ventricular mass and volume by multiple imaging modalities.^3^^,^^4^ Similarly, increased biventricular mass by cardiac magnetic resonance imaging (CMR) has been found to be increased in obese children following tetralogy of Fallot repair.^5^ Similar relationships between ventricular characteristics and body mass index (BMI) have yet to be described in the adult single ventricle population.

The long-term outcomes of obesity in Fontan are becoming clearer. Increased BMI in adult Fontan has been associated with mortality, transplant, or hospice referral^6^ and increasing weight in adulthood has been associated with Fontan failure.^7^ Elevated BMI in adult Fontan has also been associated with worse cardiovascular hemodynamics and adverse clinical outcomes including increased thromboembolic events and heart failure hospitalizations.^8^ Ventricular characteristics by CMR have also been demonstrated to prognosticate outcome in Fontan patients. CMR parameters late in Fontan physiology, particularly increased ventricular dilation, but also ventricular mass, have been associated with higher likelihood of death or transplant.^9^

In structurally normal hearts, obesity is well-known to have adverse effects on cardiovascular hemodynamics^10^^,^^11^ including increased circulating blood volume resulting in increased ventricular dilation, wall stress, and hypertrophy. In the absence of a subpulmonary ventricle in Fontan circulation, any perturbation in systemic ventricular characteristics or performance conferred by increased BMI may have substantial implications for this delicate physiology. Indeed, obesity in adult Fontan has been demonstrated to be associated with reduced peak oxygen consumption, higher filling pressures, and lower arterial oxygen saturations compared to those with overweight or normal BMI.^12^ As the relationship of increased BMI and ventricular characteristics by CMR in the adult Fontan population has yet to be described, we sought to demonstrate that, in addition to the innate changes incurred by long-term exposure to Fontan physiology, increased BMI is an independent risk factor for ventricular maladaptation, emphasizing the importance of obesity prevention in this functionally limited population.

Using the FORCE (Fontan Outcomes Registry using CMR Examinations) database, our primary objective was to compare CMR characteristics across traditional BMI categories to evaluate the single ventricle response to increased BMI in Fontan circulation. We hypothesized that, similar to structurally normal hearts, increased BMI in adult Fontan patients is associated with higher ventricular volume and mass. Secondarily, we compared associated hemodynamics by cardiac catheterization as well as clinical outcome following CMR across BMI groups to improve our understanding of the physiologic and clinical impact of overweight and obesity in Fontan patients. Finally, we stratified our analyses by ventricular morphology to evaluate for potential BMI-related adaptive differences in left, right, and mixed ventricular morphologies.

## Methods

### Force database

We performed a multi-institutional, multinational retrospective cross-sectional study using the FORCE database. FORCE is a multicenter Fontan registry involving 35 participating centers with currently more than 5,000 CMRs and over 3,500 individual Fontan patients. In addition to CMR data, FORCE includes relevant patient demographics, surgical history, clinical outcome, and other diagnostic data temporally associated within 12 months of CMR including echocardiography (ECHO), cardiopulmonary exercise testing, and cardiac catheterization. Though CMR protocols varied by institution, CMR data in FORCE are derived from processing of standard acquisitions per established single ventricle guidelines^13^^,^^14^ resulting in standardized reproducibility. Institutional variation and diverse CMR indications in adult Fontan patients inevitably result in missingness within the database, particularly for CMR characteristics not customarily obtained with every Fontan study and not needed to answer the clinical question for which the CMR was obtained (Supplemental Table 1).

This study was approved by the Institutional Review Board at Boston Children’s Hospital. Participating FORCE centers either relied on the Boston Children’s Hospital Institutional Review Board or had the study approved by their local Institutional Review Board or ethics review committee. The study proposal and this manuscript were approved by the FORCE Data Governance and Publications Committee and the FORCE Investigators. This study included all the CMR examinations in the FORCE registry as of October 2022. The data underlying this article are available from the corresponding author upon reasonable request.

### Patient selection

**Figure 1:**
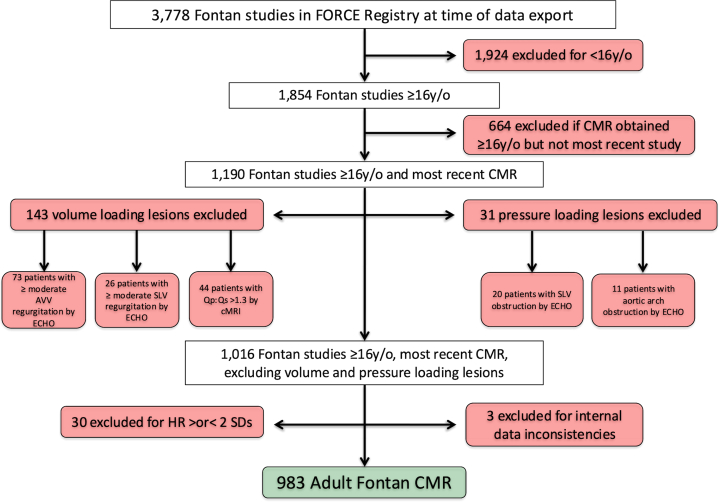
**Patient Selection Flowchart: Our Exclusion Criteria and the Number of Patients Within Each Exclusion Group** Fontan patients ≥16 years of age were included. If a patient had multiple CMR studies, we included only the most recent. Several potential perturbations of ventricular pressure and/or volume loading were excluded. The final inclusion number of the overall cohort was 983 adult Fontan studies. AVV = atrioventricular valve; CMR = cardiac magnetic resonance imaging; ECHO = echocardiogram; HR = heart rate; Qp:Qs = ratio of pulmonary to systemic blood flow; SLV = semilunar valve.

At the time of the FORCE database export, there were 3,778 CMR studies included in the registry. A comprehensive summary of CMR study inclusions and exclusions is outlined in Figure 1. Patients <16 years of age were excluded. We excluded duplicate CMR studies for the same patient, including only the most recent study, while concurrently electing to control for age at the time of CMR in our multivariable analysis. This was done to ensure our analysis accounted for the hemodynamic effects on ventricular remodeling due to longer exposure to Fontan circulation. Endeavoring to isolate the effect of BMI on ventricular characteristics, we excluded significant physiologic perturbations that might affect volume and mass. Volume loading lesions including a Qp:Qs >1.3:1 as well as moderate or more atrioventricular or semilunar valve regurgitation by ECHO were excluded. Patients with semilunar valve or aortic arch obstruction by ECHO were also excluded due to the likely effect of these lesions on ventricular mass. Following these exclusions, heart rates above or below two SDs from the mean were additionally excluded in order to reduce the per-beat variance in ventricular volume at the time of CMR. Three additional patients were excluded after identification of internal data inconsistencies. ECHO and cardiac catheterization data were included only if obtained within 12 months before or after CMR. If multiple ECHO studies or cardiac catheterizations were performed during this time frame, the closest to the date of CMR was utilized.

### Data analysis

The primary independent variable was patient BMI at the time of CMR which, to provide a pragmatic clinical approach, was organized by traditional BMI categories: underweight (BMI <18.5), normal weight (BMI 18.5-24.9), overweight (BMI 25.0-29.9), and obese (BMI ≥30.0). The primary dependent variables were CMR characteristics. Given our primary independent variable is already an indexed value, we elected to use nonindexed CMR characteristics which is consistent with previous studies investigating the impact of obesity on ventricular size and function.^15^ A noncontrast variance score test was performed to evaluate for linearity between both end-diastolic volume (EDV) and ventricular mass compared to BMI as a continuous variable and can be found in the Supplemental Figure 1.

To estimate the association between ventricular changes related to BMI with Fontan hemodynamics, a secondary analysis was performed using cardiac catheterization data as the dependent variables while BMI category remained the primary independent variable. The underweight BMI group was excluded from this analysis due to a paucity of patients within this group who underwent cardiac catheterization (N = 23). To capture the clinical significance of increased BMI in adults with Fontan, an additional analysis was performed utilizing clinical outcome data available in the FORCE registry including NYHA functional class, atrial arrhythmia, ventricular arrhythmia, diuretic requirement, and a composite outcome of heart transplantation listing or death. In univariable analysis, using BMI category as the independent variable, Mann-Whitney *U* and Kruskal-Wallis tests were used for binomial and nonordinal categorical variables, respectively. Ordinal categorical and continuous variables were compared using Spearman correlation.

Multivariable models for CMR characteristics were created for the overall cohort and stratified by ventricular morphology, adjusting for age at CMR, sex, height, ventricular ejection fraction, NYHA functional class, and diuretic use. The model for the overall cohort was additionally adjusted for ventricular morphology. Variable selection was chosen based on clinical relevance and plausible physiologic mechanisms. Several variables with significant association to BMI in univariable analysis were not included in the multivariable analysis as they were felt to be colinear with those already included in the model, particularly age at CMR. Moreover, collinearity testing was performed and was negative.

Additional analyses comparing BMI category to CMR characteristics, hemodynamics by cardiac catheterization, and clinical outcome data were performed while stratifying by ventricular morphology groups, defined as morphologic left ventricle (MLV), morphologic right ventricle (MRV), and mixed morphology (MM). Only MLV and MRV were included in the cardiac catheterization and clinical outcome stratified analyses to accomplish a direct left vs right morphologic comparison. Joint models evaluating interaction between BMI category and ventricular morphology for CMR characteristics were additionally performed (Supplemental Figure 2).

Statistical analysis was performed using Statistical Package for the Social Sciences version 29.0 (IBM), Stata/SE 15.0 (StataCorp), and R version 3.6.1.

## Results

### Patient demographics

CMR studies from 983 unique Fontan patients were included. Patient demographics stratified by BMI category are summarized in Table 1. The majority of the overall cohort were normal weight (55.1%) or overweight (23.3%). Fewer patients were obese (15.4%) and only 6.2% of patients were underweight. Of the total cohort, 41.5% were female with no significant differences in sex across BMI categories. Nearly half of patients in the overall cohort had a MLV (N = 467, 47.5%) while 353 (35.9%) had MRV and 163 (16.6%) had MM. The most common cardiac diagnoses were hypoplastic left heart syndrome (22.8%), tricuspid atresia (22.7%), double outlet right ventricle (12.8%), and double inlet left ventricle (12.3%). There were no significant differences in cardiac diagnosis or ventricular morphology across BMI categories. The proportion of patients with an extracardiac Fontan decreased with increasing BMI category (45.9% underweight, 17.9% obese; *P* < 0.001). The median age at the time of CMR in the overall cohort was 21.9 years and the median time in Fontan circulation was 18.0 years. Age at CMR and time in Fontan circulation at CMR were both significantly higher in overweight and obese patients compared to normal or underweight patients (*P* < 0.001 for each). At the time of CMR, patients who were underweight had lower mean arterial pressure (81.8 mm Hg) compared to the remainder of the cohort (mean 84.7 mm Hg for the remaining BMI categories).

**Table 1:** Patient Demographics in the Overall Cohort and Stratified by BMI Category

	Overall Cohort (N = 983)	Underweight (BMI <18.5) (n = 61, 6.2%)	Normal Weight (BMI 18.5-24.9) (n = 542, 55.1%)	Overweight (BMI 25.0-29.9) (n = 229, 23.3%)	Obese (BMI ≥30.0) (n = 151, 15.4%)	*P* Value
Patient demographics						
Female	408 (41.5%)	21 (34.4%)	226 (41.7%)	99 (43.2%)	62 (41.1%)	0.538
Age at Fontan (y)	2.9 (2.0-4.9)	3.0 (2.0-4.9)	2.8 (2.0-4.3)	2.9 (1.9-5.3)	3.2 (2.2-5.5)	0.039
Cardiac diagnosis						
Hypoplastic left heart syndrome	224 (22.8%)	10 (16.4%)	134 (24.7%)	54 (23.6%)	26 (17.2%)	0.376
Tricuspid atresia	223 (22.7%)	14 (23.0%)	127 (23.4%)	51 (22.3%)	31 (20.5%)	
Double outlet right ventricle	126 (12.8%)	7 (11.5%)	65 (12.0%)	31 (13.5%)	23 (15.2%)	
Double inlet left ventricle	121 (12.3%)	5 (8.2%)	62 (11.5%)	27 (11.8%)	27 (17.9%)	
Pulmonary atresia with intact	71 (7.2%)	5 (8.2%)	39 (7.2%)	18 (7.9%)	9 (6.0%)	
Ventricular septum						
Atrioventricular septal defect	56 (5.7%)	5 (8.2%)	31 (5.7%)	11 (4.8%)	9 (6.0%)	
Ebstein anomaly or other	53 (5.4%)	6 (9.8%)	30 (5.5%)	13 (5.7%)	4 (2.6%)	
Hypoplastic right ventricle						
Complex two ventricle anatomy	30 (3.1%)	2 (3.3%)	15 (2.8%)	5 (2.2%)	8 (5.3%)	
Mitral atresia	19 (1.9%)	1 (1.6%)	10 (1.9%)	7 (3.0%)	1 (0.7%)	
Double inlet right ventricle	10 (1.0%)	0 (0.0%)	4 (0.7%)	4 (1.7%)	2 (1.3%)	
Other or unknown	50 (5.1%)	6 (9.8%)	25 (4.6%)	8 (3.5%)	11 (7.3%)	
Dominant ventricular morphology						
Morphologic left ventricle	467 (47.5%)	32 (52.4%)	253 (46.7%)	105 (45.9%)	76 (50.3%)	0.940
Morphologic right ventricle	353 (35.9%)	17 (27.9%)	201 (37.1%)	93 (40.6%)	43 (28.5%)	
Mixed morphology	163 (16.6%)	12 (19.7%)	88 (16.2%)	31 (13.5%)	32 (21.2%)	
Fontan type at cardiac MRI						
Atriopulmonary	107 (10.9%)	5 (8.2%)	50 (9.2%)	34 (14.8%)	18 (11.9%)	<0.001
Lateral tunnel	558 (56.8%)	25 (41.0%)	308 (56.8%)	132 (57.6%)	93 (61.6%)	
Extracardiac	255 (25.9%)	28 (45.9%)	152 (28.1%)	48 (21.0%)	27 (17.9%)	
Other or unknown	63 (6.4%)	3 (4.9%)	32 (5.9%)	15 (6.6%)	13 (8.6%)	
Cardiac MRI characteristics						
Age at CMR (y)	21.9 (18.2-27.7)	18.2 (16.9-20.3)	20.8 (17.8-25.3)	24.7 (20.4-31.2)	26.0 (19.6-32.7)	<0.001
Time in Fontan circulation at CMR (y)	18.0 (14.8-22.4)	15.1 (13.2-16.5)	17.4 (14.7-21.2)	19.9 (15.9-24.0)	21.2 (16.1-26.0)	<0.001
Weight at CMR (kg)	66.7 (56.7-79.0)	48.0 (44.0-52.0)	60.1 (54.1-67.6)	76.2 (69.0-82.3)	95.3 (85.5-104.7)	<0.001
Height at CMR (cm)	168.0 (160.0-174.0)	167.4 (161.3-172.5)	167.8 (160.0-174.0)	167.0 (161.0-174.0)	169.0 (162.0-173.5)	0.216
Heart rate (beats/min)	73.0 (64.0-82.0)	78.0 (61.8-79.3)	72.0 (63.0-82.0)	72.0 (63.0-80.8)	71.0 (60.0-81.0)	0.120
Mean arterial pressure (mm Hg)	84.5 (77.6-91.2)	81.8 (76.5-85.8)	84.3 (77.5-91.0)	82.9 (76.2-89.5)	86.8 (78.9-92.8)	0.027

All categorical variables expressed as N (%). All continuous variables expressed as median (IQR).

Categorical binomial and nonordinal variables were compared to BMI category using Mann-Whitney *U* and Kruskal-Wallis tests, respectively.

Continuous variables were compared to BMI category using Spearman correlation.

BMI = body mass index; CMR = cardiac magnetic resonance imaging; MRI = magnetic resonance imaging.

### CMR characteristics

CMR characteristics for the overall cohort stratified by BMI category are displayed in Table 2. EDV and end systolic volume were both significantly increased in Fontan patients in higher BMI categories. EDV for patients with normal BMI was 159.6 mL compared to 187.0 mL in obese patients (*P* < 0.001). A similar trend was seen for end systolic volume (73.0 mL for normal BMI vs 89.0 mL in obese BMI; *P* < 0.001). Ventricular mass was also significantly higher with increasing BMI category. Normal BMI patients had a ventricular mass of 92.0 g compared to 118.0 g in obese Fontan patients (*P* < 0.001). The percentage of Fontan patients with identifiable venovenous collaterals by CMR increased with higher BMI category. Only one (1.6%) of underweight patients had venovenous collaterals present while they were identified in 34 (22.5%) of obese Fontan patients (*P* < 0.001). In contrast, the presence of aortopulmonary collaterals did not vary significantly across BMI category.

**Table 2:** CMR Characteristic Stratified by BMI Category (N = 983)

	Underweight (BMI <18.5) (n = 61, 6.2%)	Normal Weight (BMI 18.5-24.9) (n = 542, 55.1%)	Overweight (BMI 25.0-29.9) (n = 229, 23.3%)	Obese (BMI ≥30.0) (n = 151, 15.4%)	*P* Value
End-diastolic volume (mL)	134.5 (109.6-169.5)	159.6 (126.0-208.1)	171.95 (132.0-221.7)	187.0 (146.2-227.0)	<0.001
End systolic volume (mL)	60.2 (48.5-91.2)	73.0 (53.0-106.3)	78.2 (54.1-110.2)	89.0 (63.0-117.0)	<0.001
Stroke volume (mL)	72.0 (58.4-87.3)	84.7 (69.0-101.1)	88.4 (72.4-111.5)	94.0 (78.0-111.0)	<0.001
Ventricular mass (g)	82.0 (57.8-112.9)	92.0 (69.0-123.6)	102.0 (78.7-142.0)	118.0 (87.1-151.9)	<0.001
Mass/Volume ratio (g/mL)	0.55 (0.46-0.73)	0.54 (0.43-0.72)	0.59 (0.44-0.81)	0.60 (0.45-0.80)	0.020
Ventricular ejection fraction (%)	53.0 (47.1-58.0)	53.1 (46.4-59.3)	53.8 (46.6-59.6)	51.1 (44.9-57.1)	0.180
Ventricular vascular coupling ratio	0.89 (0.73-1.13)	0.89 (0.68-1.16)	0.86 (0.68-1.11)	0.94 (0.75-1.22)	0.308
Positive for late gadolinium enhancement	11 (18.0%)	97 (17.9%)	41 (17.9%)	21 (13.9%)	0.433
Fenestration or baffle leak	19 (31.2%)	156 (28.8%)	51 (22.3%)	28 (18.5%)	0.018
Presence of venovenous collaterals	1 (1.6%)	73 (13.5%)	34 (14.8%)	34 (22.5%)	<0.001
Presence of aortopulmonary collaterals	10 (16.4%)	62 (11.4%)	22 (9.6%)	12 (8.0%)	0.101

All categorical variables expressed as N (%). All continuous variables expressed as median (IQR).

Categorical binomial variables were compared to BMI category using Mann-Whitney *U* tests.

Continuous variables were compared to BMI category using Spearman correlation.

Abbreviations as in Table 1.

Notably, EDV (*P* = 0.027) and ventricular mass (*P* = 0.003) were found to be nonlinear compared to BMI as a continuous variable (Supplemental Figure 3). Despite this, primary representation of BMI as a categorized variable was maintained to provide a clinically practical conceptualization.

Multivariable analysis adjusting for age at CMR, sex, height, ventricular ejection fraction, NYHA functional class, diuretic use, and ventricular morphology was performed for EDV and ventricular mass in the overall cohort and is demonstrated in Table 3. Compared to patients with normal weight, EDV for Fontan patients with overweight and obese BMI remained significantly increased (+13.9 mL; 95% CI: 5.6-22.2; *P* = 0.001 and + 18.0 mL; 95% CI: 8.3-27.7; *P* < 0.001 for overweight and obese BMI, respectively). EDV for underweight BMI remained significantly lower than in patients with normal weight (−21.9 mL; 95% CI: −35.9 to −7.8; *P* = 0.002). Similarly, ventricular mass for patients with overweight and obese BMI remained significantly increased compared to normal weight (+13.8 g; 95% CI: 4.7-22.9; *P* = 0.003 and + 19.1 g; 95% CI: 8.7-29.6; *P* < 0.001 for overweight and obese BMI, respectively). Notably, the regression coefficients for both EDV and ventricular mass rise in a dose-related relationship with each increasing BMI category.

**Table 3:** Multivariable Analysis of EDV and Ventricular Mass in the Overall Cohort and by Ventricular Morphology

	CMR Characteristic	Underweight (BMI <18.5)		Normal Weight (BMI 18.5-24.9)		Overweight (BMI 25.0-29.9)		Obese (BMI ≥30.0)	
		Coef. (CI)	*P*	Coef. (CI)	*P*	Coef. (CI)	*P*	Coef. (CI)	*P*
Overall cohort	EDV	−21.9 (−35.9, −7.8)	0.002	-		+13.9 (5.6, 22.2)	0.001	+18.0 (8.3, 27.7)	<0.001
	Ventricular mass	−11.9 (−28.0, 4.2)	0.147	-		+13.8 (4.7,22.9)	0.003	+19.1 (8.7, 29.6)	<0.001
Morphologic left ventricle	EDV	−18.2 (−35.9, −0.6)	0.043	-		+12.6 (1.5, 23.8)	0.026	+23.2 (10.5, 36.0)	<0.001
	Ventricular mass	−2.5 (−18.5, 13.5)	0.757	-		+14.9 (5.3, 24.5)	0.003	+12.4 (1.5, 23.3)	0.026
Morphologic right ventricle	EDV	−43.2 (−72.9, −13.6)	0.004	-		+9.8 (−4.9, 24.4)	0.191	+5.5 (−13.4, 24.5)	0.567
	Ventricular mass	−40.7 (−90.4, 9.0)	0.108	-		+15.6 (−5.1, 36.3)	0.139	+22.0 (−3.6, 47.7)	0.092
Mixed morphology	EDV	−4.1 (−33.1, 24.9)	0.781	-		+33.1 (12.7, 53.4)	0.022	+24.2 (2.8, 45.5)	0.027
	Ventricular mass	−14.0 (−46.8, 18.8)	0.399	-		+9.3 (−13.4, 31.9)	0.501	+30.6 (4.9, 56.2)	0.020

Model adjusted for age at CMR, sex, height, ventricular ejection fraction, NYHA functional class, and diuretic use. Overall cohort model also adjusted for ventricular morphology.

Reference = normal weight.

EDV = end-diastolic volume; other abbreviations as in Table 1.

### Hemodynamics by cardiac catheterization

Hemodynamic data were available for 427 patients who underwent cardiac catheterization within 12 months of CMR (Table 4). As mentioned previously, underweight BMI was excluded due to a small sample size. The majority (N = 251, 58.8%) were in the normal weight BMI category while 107 (25.1%) and 69 (16.1%) patients underwent cardiac catheterization in the overweight and obese BMI groups, respectively. Systemic ventricular end-diastolic pressure was significantly higher for patients in the obese BMI category (10.5 mm Hg) compared to the normal and overweight categories (each 9.0 mm Hg; *P* = 0.006). Fontan pressure was also significantly higher for obese Fontan patients (15.0 mm Hg vs 13.0 mm Hg in both normal and overweight categories; *P* = 0.004).

**Table 4:** Cardiac Catheterization Data in the Overall Cohort Stratified by BMI Category (N = 427)

	Normal Weight (BMI 18.5-24.9) (n = 251)	Overweight (BMI 25.0-29.9) (n = 107)	Obese (BMI ≥30.0) (n = 69)	*P* Value
Systemic ventricular end-diastolic pressure (mm Hg)	9.0 (7.0-11.0)	9.0 (8.0-12.0)	10.5 (8.0-12.5)	0.006
Pulmonary capillary wedge pressure (mm Hg)	8.5 (7.0-10.5)	8.8 (7.0-10.5)	10.0 (8.0-12.0)	0.055
Left pulmonary artery mean pressure (mm Hg)	12.0 (10.0-14.3)	12.0 (10.0-14.0)	14.0 (12.8-16.0)	0.085
Right pulmonary artery mean pressure (mm Hg)	12.0 (10.0-14.0)	12.0 (10.0-14.0)	14.0 (12.5-16.0)	0.065
Fontan pressure (mm Hg)	13.0 (11.0-15.0)	13.0 (11.0-15.0)	15.0 (13.0-16.0)	0.004
Pulmonary vascular resistance (WU)	1.5 (1.0-1.9)	1.5 (1.0-2.2)	1.9 (1.2-2.3)	0.060
Cardiac index (L/min/BSA)	3.0 (2.4-3.5)	2.8 (2.4-3.4)	2.6 (2.2-3.5)	0.087
Qp:Qs	0.9 (0.8-1.0)	0.9 (0.8-1.0)	0.9 (0.8-1.0)	0.952
Oxygen saturation (%)	92.0 (89.3-95.0)	92.0 (90.8-95.0)	89.0 (87.0-93.5)	0.114

Continuous variables expressed as median (IQR).

Test used: Spearman correlation.

Underweight BMI group was excluded due to a small sample size for comparison group (N = 23).

BSA = body surface area; Qp:Qs = ratio of pulmonary to systemic blood flow; other abbreviation as in Table 1.

### Clinical outcome

Clinical outcome data were compared across BMI categories to provide a more comprehensive perspective of the impact of overweight and obesity in adults with Fontan circulation (Table 5). Median time from CMR to clinical follow-up was 5.2 years. In the overall cohort, worse NYHA functional class, atrial arrhythmia, and diuretic requirement were all significantly associated with increased BMI category (*P* < 0.001 for all). Ventricular arrhythmia and a composite outcome of heart transplant listing and death were not associated with BMI status in the overall cohort.

**Table 5:** Clinical Outcome Following CMR

	Overall Cohort				
	Underweight (BMI <18.5)	Normal Weight (BMI 18.5-24.9)	Overweight (BMI 25.0-29.9)	Obese (BMI ≥30.0)	*P* Value
NYHA functional class					
I	41 (73.2%)	393 (73.4%)	134 (60.6%)	87 (61.7%)	<0.001
II	14 (25.0%)	110 (20.6%)	68 (30.8%)	36 (25.5%)	
III	1 (1.8%)	25 (4.7%)	16 (7.2%)	15 (10.7%)	
IV	0 (0.0%)	7 (1.3%)	3 (1.4%)	3 (2.1%)	
Atrial arrhythmia	10 (16.4%)	138 (25.5%)	99 (43.2%)	61 (40.4%)	<0.001
Ventricular arrhythmia	2 (3.3%)	61 (11.3%)	26 (11.4%)	11 (7.3%)	0.935
Diuretic requirement	13 (21.3%)	139 (25.7%)	98 (42.8%)	61 (40.4%)	<0.001
Heart transplant listing or death	4 (6.6%)	32 (5.9%)	21 (9.2%)	12 (8.0%)	0.174

Morphologic Left Ventricle					
NYHA functional class					
I	21 (70.0%)	183 (73.2%)	57 (54.8%)	40 (58.0%)	0.011
II	8 (26.7%)	58 (23.2%)	37 (35.6%)	20 (29.0%)	
III	1 (3.3%)	8 (3.2%)	9 (8.6%)	7 (10.1%)	
IV	0 (0.0%)	1 (0.4%)	1 (1.0%)	2 (2.9%)	
Atrial arrhythmia	6 (18.8%)	70 (27.7%)	50 (47.2%)	33 (43.4%)	<0.001
Ventricular arrhythmia	1 (3.1%)	27 (10.7%)	15 (14.2%)	6 (7.9%)	0.539
Diuretic requirement	7 (21.9%)	56 (22.1%)	39 (36.8%)	28 (36.8%)	0.001
Heart transplant listing or death	0 (0.0%)	10 (4.0%)	6 (5.7%)	6 (7.9%)	0.061

Morphologic Right Ventricle					
NYHA functional class					
I	11 (78.6%)	141 (71.6%)	58 (67.5%)	31 (77.5%)	0.829
II	3 (21.4%)	37 (18.8%)	21 (24.4%)	6 (15.0%)	
III	0 (0%)	13 (6.6%)	5 (5.8%)	3 (7.5%)	
IV	0 (0%)	6 (3.0%)	2 (2.3%)	0 (0.0%)	
Atrial arrhythmia	1 (5.9%)	47 (23.4%)	33 (35.9%)	14 (32.6%)	0.005
Ventricular arrhythmia	1 (5.9%)	20 (10.0%)	7 (7.6%)	3 (7.0%)	0.576
Diuretic requirement	3 (17.6%)	61 (30.3%)	47 (51.1%)	19 (44.2%)	<0.001
Heart transplant listing or death	1 (5.9%)	20 (10.0%)	12 (13.0%)	2 (4.7%)	0.981

Median time from CMR to follow-up: 5.2 years.

All categorical variables expressed as N (%).

^c^Categorical binomial and ordinal variables were compared to BMI category using Mann-Whitney *U* test and Spearman correlation, respectively.

Abbreviations as in Table 1.

### Stratification by ventricular morphology

**Figure 2:**
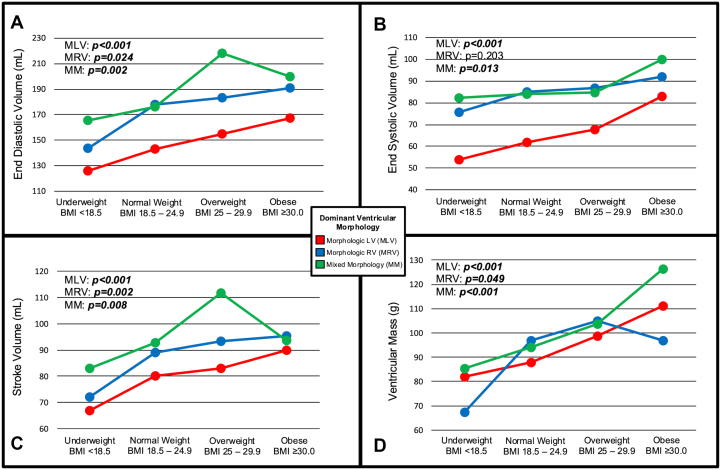
**CMR Characteristics Vs BMI Categories Stratified by Ventricular Morphology** Association of CMR characteristics related to BMI category stratified by ventricular morphology: MLV (N = 467), MRV (N = 353), MM (N = 163). Median values of end-diastolic volume (A), end systolic volume (B), stroke volume (C), and ventricular mass (D) are displayed. BMI = body mass index; MLV = morphologic left ventricle; MM = mixed morphology; MRV = morphologic right ventricle; other abbreviation as in Figure 1.

To assess the potential for disparate morphologic ventricular responses to obesity, CMR characteristics were compared across BMI categories stratified by Fontan patients with MLV, MRV, and MM. Stratified comparisons for EDV, end systolic volume, stroke volume, and ventricular mass are summarized in Figure 2. Absolute EDV was higher for patients with MRV and MM compared to MLV in all BMI categories and EDV significantly increased with higher BMI categories for all ventricular morphologies (*P* < 0.001, *P* = 0.024, *P* = 0.002 for MLV, MRV, and MM, respectively). All ventricular morphologies also demonstrated significantly increased ventricular mass in higher BMI categories (*P* < 0.001, *P* = 0.049, and *P* < 0.001, for MLV, MRV, and MM, respectively). Stratified comparisons for venovenous collaterals were also performed. A larger percentage of Fontan patients with detectable venovenous collaterals by CMR was demonstrated in Fontan patients with MLV in increased BMI categories (*P* = 0.001) with >25% of obese Fontan patients with MLV having radiographically discernable venovenous collaterals. There was no significant difference in the presence of venovenous collaterals across BMI categories for patients with MRV.

When testing for interaction between BMI category and ventricular morphology, among CMR characteristics, only the model for EDV was significant (*P* = 0.041.

Multivariable analysis was performed for EDV and ventricular mass stratified by ventricular morphology (Table 3). Fontan patients with MLV followed trends seen in the overall cohort. Compared to patients with normal BMI, patients with MLV had significantly increased EDV and ventricular mass in overweight BMI (+12.6 mL; 95% CI: 1.5-23.8; *P* = 0.026 and + 14.9 g; 95% CI: 5.3-24.5; *P* = 0.003 for EDV and ventricular mass, respectively) and obese BMI (+23.2 mL; 95% CI: 10.5-36.0; *P* < 0.001 and + 12.4 g; 95% CI: 1.5-23.3; *P* = 0.026 for EDV and ventricular mass, respectively). Compared to normal BMI, significantly lower EDV was demonstrated in underweight patients with MLV (−18.2 mL; 95% CI: −35.9 to −0.6; *P* = 0.043). Correlation coefficients again generally demonstrated a dose-dependent BMI effect for both EDV and ventricular mass with each increasing BMI category. Underweight Fontan patients with MRV had significantly lower EDV compared to normal BMI (−43.2 mL; 95% CI: −72.9 to −13.6; *P* = 0.004), however, overweight and obese Fontan patients with MRV did not have significantly increased EDV or ventricular mass compared to normal BMI. In Fontan patients with MM, EDV was significantly higher in the overweight and obese BMI groups compared to normal BMI (*P* = 0.022, *P* = 0.027, respectively), however, ventricular mass was not found to be consistently significantly different across BMI categories.

Results from hemodynamics by cardiac catheterization were also stratified by ventricular morphology, the summary of which can be found in Table 6. Similar to the nonstratified cardiac catheterization comparison, underweight BMI was excluded due to small sample size (N = 17). Patients with MM were also not included as to directly compare left vs right ventricular morphologies. A similar number of MLV (N = 176) and MRV (N = 187) patients had available cardiac catheterization data, and, the distribution of patients within each BMI category was comparable to the nonstratified cardiac catheterization analysis. In Fontan patients with MLV, median values for ventricular end-diastolic pressure, pulmonary capillary wedge pressure, left and right pulmonary artery mean pressures, Fontan pressure, and pulmonary vascular resistance were all significantly higher with increasing BMI category (*P* < 0.05 for all values). Percent oxygen saturation was significantly lower in higher BMI categories (*P* = 0.044). No differences in hemodynamic data were detected across BMI groups in Fontan patients with MRV.

**Table 6:** Cardiac Catheterization Data in Left and Right Ventricular Morphologies Stratified by BMI Category (N = 363)

	Normal Weight (BMI 18.5-24.9) (n = 213: MLV = 102, MRV = 111)	Overweight (BMI 25.0-29.9) (n = 95: MLV = 42, MRV = 53)	Obese (BMI ≥30.0) (n = 55: MLV = 32, MRV = 23)	*P* Value
Morphologic left ventricle N = 176				
Systemic ventricular end-diastolic pressure (mm Hg)	8.0 (5.9-10.0)	9.0 (8.0-10.5)	11.0 (8.4-13.0)	0.001
Pulmonary capillary wedge pressure (mm Hg)	8.0 (6.1-10.0)	9.0 (7.0-10.0)	10.5 (8.0-12.0)	0.002
Left pulmonary artery mean pressure (mm Hg)	11.5 (9.0-13.8)	11.0 (10.0-13.0)	14.0 (13.0-16.0)	0.007
Right pulmonary artery mean pressure (mm Hg)	11.5 (10.0-14.0)	12.0 (10.0-14.0)	14.0 (13.0-16.0)	<0.001
Fontan pressure (mm Hg)	12.5 (11.0-15.0)	13.0 (11.0-15.0)	15.0 (14.0-16.0)	0.002
Pulmonary vascular resistance (WU)	1.40 (1.00-1.87)	1.80 (1.10-2.75)	1.90 (1.20-2.20)	0.023
Cardiac index (L/min/BSA)	3.02 (2.40-3.79)	2.95 (2.28-3.53)	2.61 (2.20-3.28)	0.172
Qp:Qs	0.9 (0.8-1.0)	0.9 (0.8-1.0)	0.9 (0.8-1.0)	0.805
Oxygen saturation (%)	93.0 (90.0-95.0)	92.0 (90.8-94.3)	91.0 (88.0-93.5)	0.044
Morphologic right ventricle N = 187				
Systemic ventricular end-diastolic pressure (mm Hg)	9.0 (8.0-11.3)	9.5 (7.0-12.8)	11.0 (9.3-14.0)	0.161
Pulmonary capillary wedge pressure (mm Hg)	9.5 (7.0-10.5)	8.0 (6.8-10.5)	9.3 (8.0-11.0)	0.920
Left pulmonary artery mean pressure (mm Hg)	13.0 (10.0-15.0)	12.0 (10.0-14.0)	14.0 (12.0-16.5)	0.724
Right pulmonary artery mean pressure (mm Hg)	13.0 (10.0-15.0)	12.0 (10.0-14.0)	13.5 (12.3-16.5)	0.852
Fontan pressure (mm Hg)	13.0 (11.0-15.0)	13.0 (11.8-15.0)	15.0 (12.8-18.3)	0.221
Pulmonary vascular resistance (WU)	1.54 (1.08-1.95)	1.30 (1.04-1.96)	1.75 (1.50-2.15)	0.598
Cardiac index (L/min/BSA)	3.00 (2.50-3.30)	2.80 (2.40-3.40)	2.95 (2.28-3.80)	0.872
Qp:Qs	0.9 (0.8-1.0)	1.0 (0.8-1.0)	0.8 (0.7-1.0)	0.580
Oxygen saturation (%)	92.0 (86.0-94.0)	93.0 (91.0-95.0)	89.0 (86.8-92.0)	0.775

Continuous variables expressed as median (IQR).

Test used: Spearman correlation.

Underweight BMI group excluded due to a small sample size for comparison group (N = 17).

MLV = morphologic left ventricle; MRV = morphologic right ventricle; other abbreviations as in Tables 1 and 4.

Stratified clinical outcome data (Table 5) were consistent with the overall cohort except for NYHA functional class which did not have a significant association with increased BMI in patients with MRV.

## Discussion

### BMI is an independent risk factor for ventricular maladaptation in adult Fontan

This study demonstrates that ventricular adaptations in volume and mass incurred by exposure to Fontan circulation are compounded by increased BMI (Central Illustration). While increased left ventricular volume and mass are known to be associated with obesity in the non-adult congenital heart disease (ACHD) population^3^^,^^4^, our study is the first to demonstrate an analogous relationship in a large, multi-institutional adult Fontan cohort. These findings are particularly impactful given others have demonstrated increases in ventricular volume and mass in Fontan to be associated with poor outcomes such as physiologic failure and death/transplant.^9^ While longitudinal ventricular changes and Fontan failure are known to occur as an innate result of long-term Fontan circulation,^16^^,^^17^ significant BMI-related differences in both EDV and ventricular mass persisted in our multivariable analysis that controlled for age at the time of CMR, NYHA functional class, and diuretic use, further underscoring the independent association of increased BMI with ventricular remodeling in adult Fontan patients. When stratified by ventricular morphology, obesity appears to disproportionately affect Fontan patients with MLV and also impact hemodynamics by cardiac catheterization.

**Central Illustration:**
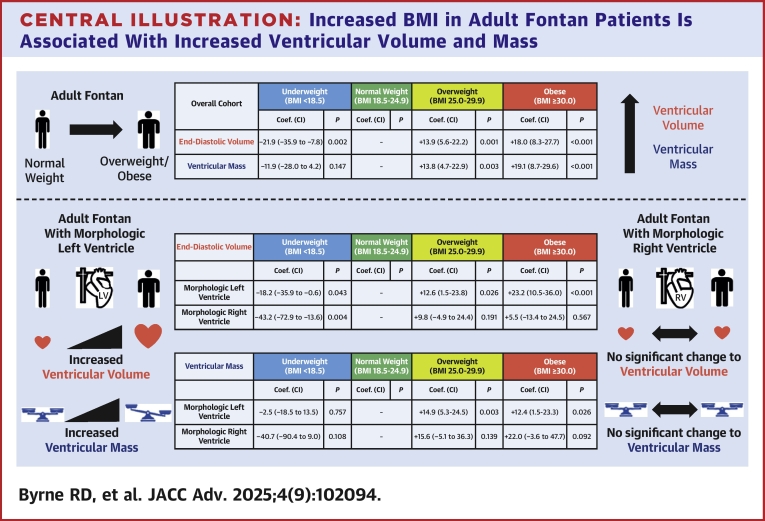
**Increased BMI in Adult Fontan Patients Is Associated With Increased Ventricular Volume and Mass** Utilizing a multi-institutional, multinational Fontan CMR database (FORCE), increased BMI in adult Fontan patients is associated with higher ventricular volume and mass. When stratified by ventricular morphology, significant multivariable associations are similar for Fontan patients with morphologic left ventricle and were largely not identified in patients with morphologic right ventricle. Abbreviation as in Figure 1.

## Altered Fontan hemodynamics and clinical outcomes related to BMI

Among many factors, one tenet of successful Fontan physiology involves reasonably low intracardiac filling pressures. There are a variety of mechanisms that can lead to increased ventricular end-diastolic pressure, including systemic hypertension, aortic arch obstruction, semilunar or atrioventricular valve disease, systolic or diastolic ventricular dysfunction, aortopulmonary collaterals, pericardial disease, and arrhythmia. Our study, and others,^8^ suggests increased BMI may be another hemodynamically impactful element to consider and, importantly, potentially modify.

In the absence of a subpulmonary pumping chamber, elevated intracardiac pressures obligate even higher Fontan pressures to maintain passive forward flow and cardiac output. It is possible that other obesity-related risk factors such as obstructive sleep apnea additionally contribute to increased Fontan pressure. Indeed, higher Fontan pressures in our study were associated with increased BMI and, in the literature, are known to be associated with end-organ dysfunction.^18^, ^19^, ^20^, ^21^ Association in our study of a higher percentage of radiographically identifiable venovenous collaterals in obese Fontan patients are corroboratory given angiogenesis of these vessels are a likely physiologic response to increased Fontan pressures. Given ACHD patients are known to adapt to underlying physiologic limitations despite reductions in objective functional capacity^22^, these hemodynamic changes may be occurring even in “asymptomatic” Fontan patients, particularly in those with MLV.

Despite association with increased BMI and higher ventricular pressures by cardiac catheterization, ventricular ejection fraction by CMR remained preserved, even in higher BMI groups. These data suggest physiology akin to the metabolic heart disease phenogroup seen in the heart failure with preserved ejection fraction population^23^ wherein obesity is common and diastolic dysfunction predominates. Ventricular diastolic dysfunction is a recognized failure phenotype in patients with Fontan^24^^,^^25^ and it is particularly noteworthy that the BMI-related hemodynamic changes in our study were primarily driven by patients with MLV, drawing parallels to obesity-related changes in the systemic left ventricle in the structurally normal, biventricular heart failure with preserved ejection fraction archetype. Increased BMI in our study was also associated with adult Fontan outcomes of worse NYHA functional class, atrial arrhythmia, and diuretic requirement, all of which bolster clinical similarities to the typical diastolic heart failure phenotype in structurally normal hearts.

### Potential mechanisms for disparate associations regarding ventricular morphology

In adult Fontan patients with MLV, our analysis demonstrated what is already known about the left ventricular response to obesity in the structurally normal heart. However, this was not true for Fontan patients with MRV. While patients with MRV had larger absolute EDV in all BMI groups compared to MLV as well as associations of higher ventricular volumes and mass with increased BMI in univariable analysis, associations of CMR characteristics with BMI in the stratified multivariable analysis were in general consistently absent. Questions naturally arise regarding why this distinction exists, particularly whether there may be inherent remodeling differences in response to increased BMI in systemic morphologic left vs right ventricles in Fontan circulation.

Although our study is not designed to elucidate an explanation, there are some signals toward morphologic ventricular remodeling differences in the literature. Compared to Fontan patients with MLV, those with MRV have increased CMR markers of diffuse myocardial fibrosis including higher T1 relaxometry and extracellular volume and were additionally found to have relatively decreased myocardial contractility.^26^ Similarly, reduced global longitudinal strain by ECHO has been identified in overweight and obese Fontan patients with MRV but this association was not identified in patients with MLV.^27^ Additionally, disparate adaptation in ventricular morphology has been demonstrated in relation to other physiologic stressors such as increased afterload. In a study comparing hypertrophy-signaling pathways in obstructed right and left ventricles,^28^ compared to hypertrophic left ventricles, right ventricular hypertrophy was associated with down-regulation in genes improving contractile efficiency and up-regulation of genes contributing to a maladaptive hypertrophic phenotype such as FGF-2 and TGF-β. To further our understanding of morphologic ventricular remodeling differences in response to obesity, future studies aimed at identifying both genomic and macro level myocardial differences in MLV and MRV are needed.

### Obesity in the adult fontan

While lower in prevalence than the general population, overweight and obesity in Fontan is quite high, currently affecting approximately one-third of adult survivors.^8^^,^^29^ In our cohort, overweight/obesity was 38.7%. Obesity in Fontan is likely a result of multiple factors including excessive caloric intake and genetic predisposition, though it is probable that limitations in activity additionally play a role. Reductions in Fontan peak VO2 are often due to an inability to augment preload and/or as a result of chronotropic incompetence which leads to early exertional symptoms in many Fontan patients, often limiting caloric expenditure. A restriction in activity may also come as a result of fears regarding worsening baseline health status or even sudden cardiac death. These concerns may originate from the patient, their family members, or even from members of the patient’s medical care team. However, recent data highlight the benefits of exercise in patients with chronic heart failure^30^ and exercise is generally regarded as beneficial to adult Fontan physiology.^31^, ^32^, ^33^ Thus, one physiologically beneficial means to accomplish obesity prevention in Fontan would be to change the Fontan/exercise narrative and add exercise recommendations, rather than restrictions, for our adult Fontan patients.

### Study Limitations

Inherent limitations exist with a retrospective study which can only provide association as opposed to causal relationship between increased BMI, ventricular characteristics, Fontan hemodynamics, and clinical outcome data. Although CMR data in FORCE were derived from a standardized process, variation by institution in obtaining and interpreting CMR, ECHO, and cardiac catheterization data may exist including resulting in missingness within the database for nonuniformly obtained variables that depend on the indication and/or clinical question. Thus, the reliance on reported values within the FORCE database is a consideration.

It is challenging to differentiate increased BMI related to adiposity from that due to retained fluid such as in failing Fontan physiology, which is more likely to occur over time. However, age at the time of CMR, NYHA functional class, and diuretic use were all included in our multivariable model, in which, ventricular mass and EDV remained significantly associated with increased BMI, further emphasizing the independent impact of BMI on ventricular maladaptation in adults with Fontan circulation.

## Conclusions

Increased BMI in adult Fontan patients is associated with increased ventricular mass and volume as well as worse hemodynamics by cardiac catheterization. These findings are particularly evident for patients with MLV. In a physiology with limited therapeutic targets, it is important that we identify modifiable Fontan risk factors such as obesity, and as pediatric and ACHD cardiologists, we should actively promote obesity prevention in our Fontan patients.

Perspectives **COMPETENCY IN MEDICAL KNOWLEDGE 1:** Similar to obesity-related left ventricular maladaptation in structurally normal hearts, in adult Fontan patients with MLV, increased BMI is associated with larger ventricular volume and mass.

**COMPETENCY IN MEDICAL KNOWLEDGE 2:** Association with increased BMI and higher ventricular end-diastolic pressure, NYHA functional class, diuretic use, and atrial arrhythmia in adult Fontan patients is comparable to that seen in the metabolic heart disease phenogroup of the heart failure with preserved ejection fraction population and are undesirable sequelae in an already tenuous physiology.

**TRANSLATIONAL OUTLOOK 1:** Discrepant associations of BMI and ventricular characteristics in adult Fontan patients with morphologic left and right ventricles raise questions related to remodeling differences and should be the subject of future scholarly work.

**TRANSLATIONAL OUTLOOK 2:** Obesity is a modifiable risk factor, and future investigations may utilize ventricular characteristics and invasive hemodynamics as dependent variables related to avoidance of obesity or intentional weight loss in obese Fontan patients.

## Funding support and author disclosures

The FORCE registry is supported by Additional Ventures and Evan’s Heart Fund. Authors Dr. Alsaied, Dr. Rathod, and Dr. Files (a FORCE Investigator) have research grant support from Mezzion Pharmaceuticals as primary investigators of the Fontan Udenafil Exercise Longitudinal Assessment-2 (FUEL-2) Trial (NCT #NCT05918211). The authors have reported that they have no relationships relevant to the contents of this paper to disclose.
