# Supplementary material for: Increased Body Mass Index and Ventricular Cardiac Magnetic Resonance Characteristics in Adults With Fontan Circulation
Source: JACC Adv. 2025 Aug 25;4(9):102094. doi: 10.1016/j.jacadv.2025.102094 (PMC12398853; doi:10.1016/j.jacadv.2025.102094)
Supplement: Supplemental_Appendix [file mmc1.docx]

| **Supplemental Table 1: Data Missingness** | | | |
| --- | --- | --- | --- |
| **CMR Data** | | **Cardiac Catheterization Data** | |
| **Variable** | **Missingness (%)** | **Variable** | **Missingness (%)** |
| End diastolic volume (mL) | 3.8 | Systemic Ventricular End Diastolic Pressure (mmHg) | 34.7 |
| End systolic volume (mL) | 4 | Pulmonary Capillary Wedge Pressure (mmHg) | 21.9 |
| Stroke volume (mL) | 4 | Left Pulmonary Artery Mean Pressure (mmHg) | 25.4 |
| Ventricular mass (g) | 33.1 | Right Pulmonary Artery Mean Pressure (mmHg) | 32.1 |
| Mass/Volume Ratio (g/mL) | 33.1 | Fontan Pressure | 8.6 |
| Ventricular ejection fraction (%) | 2.3 | Pulmonary Vascular Resistance (WU) | 29.4 |
| Ventricular vascular coupling ratio | 4 | Cardiac Index (L/min/BSA) | 20.6 |
| Positive for late gadolinium enhancement | 29.7 | Qp:Qs | 22.3 |
| Fenestration or baffle leak | 13 | Oxygen Saturation (%) | 43.4 |
| Presence of venovenous collaterals | 27.3 |  | |
| Presence of aortopulmonary collaterals | 23.5 |  |  |

^a^ Clinical data missingness included 3% for NYHA Class. All other clinical data missingness: 0%.

^b^ CMR = Cardiac magnetic resonance imaging. Qp:Qs = Ratio of pulmonary to systemic blood flow.

**Supplemental Figure 1: End Diastolic Volume vs. Body Mass Index as a Continuous Variable**

*
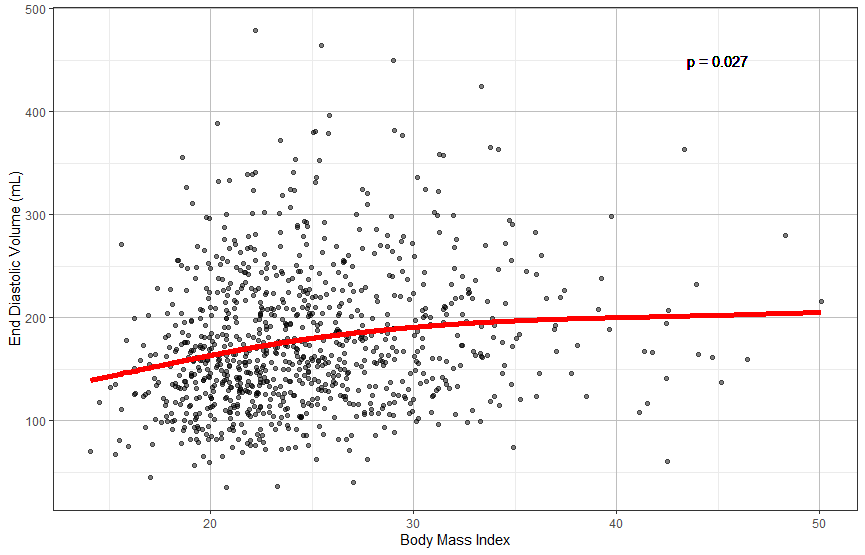
*

Caption: Supplemental Figure 1 demonstrates non-linearity of EDV with BMI as a continuous variable. Test used = non-contrast variance score test. BMI = Body mass index. EDV = End diastolic volume.

**Supplemental Figure 2: Ventricular Mass vs. Body Mass Index as a Continuous Variable**

*
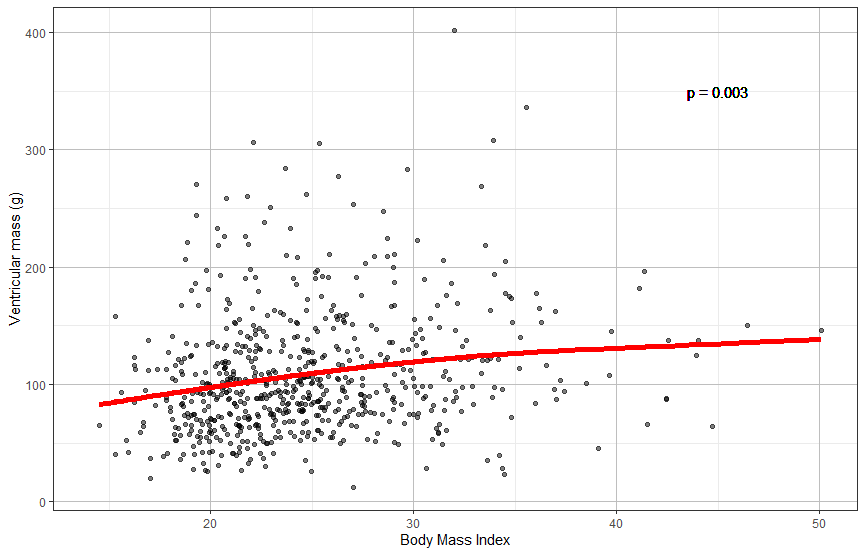
*

Caption: Supplemental Figure 2 demonstrates non-linearity of ventricular mass with BMI as a continuous variable. Test used = non-contrast variance score test. BMI = Body mass index.

**Supplemental Figure 3: Interaction of BMI Category and Ventricular Morphology for EDV**


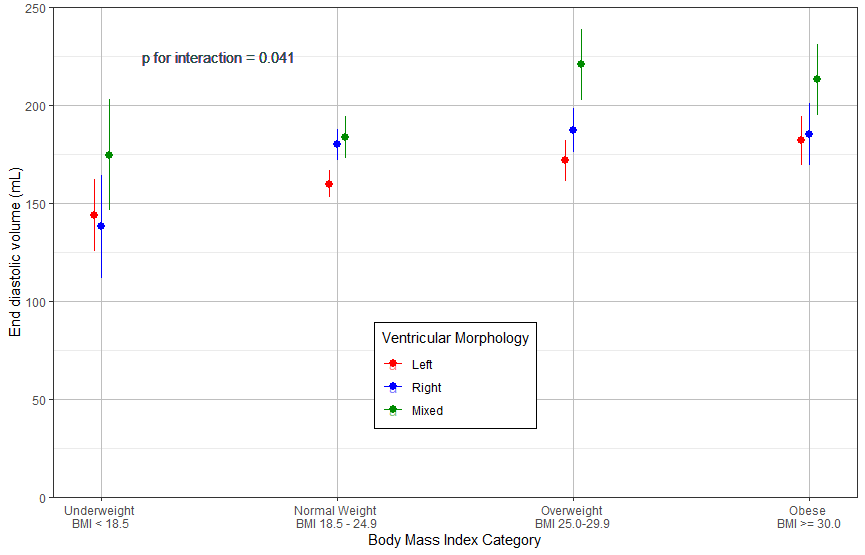


Caption: Supplemental Figure 3 demonstrates plotted data from the end diastolic volume analysis assessing interaction of BMI category with ventricular morphology. BMI = Body mass index. EDV = End diastolic volume.

| **FORCE Investigators** | |
| --- | --- |
|  |  |
| **Co-Author** | **Hospital** |
| Aggarwal, M | Department of Pediatrics, St. Louis Children's Hospital, St. Louis, MO |
| Alsaied, T | The Heart and Vascular Institute, UPMC Children's Hospital of Pittsburgh, Pittsburgh, PA |
| Dorfman, AL | Congenital Heart Center, CS Mott Children's Hospital, Ann Arbor, MI |
| Doshi, A | Department of Pediatric and Congenital Cardiology, John Hopkins Hospital, Baltimore, MD |
| Files, MD | Division of Pediatric Cardiology, Seattle Children’s Hospital, Seattle, WA |
| Fogel, M | Division of Cardiology, The Children's Hospital of Philadelphia, Pennsylvania, PA |
| Hedge, S | Division of Pediatric Cardiology, Rady Children's Hospital, San Diego, CA |
| Hoyer, A | Division of Pediatric Cardiology, University of Arizona, Tucson, AZ |
| Johnson, T | Division of Pediatric Cardiology, Riley Hospital for Children, Indianapolis, IN |
| Krishnamurthy, R | Department of Radiology, Nationwide Children's Hospital, Columbus, OH |
| Lam, CZ | Department of Diagnostic and Interventional Radiology, The Hospital for Sick Children, Toronto, Canada |
| Loke, Y | Division of Cardiology, Children's National Hospital, Washington DC |
| Marsden, AL | Department of Bioengineering & Pediatrics, Stanford University, Palo Alto, CA |
| Muthurangu, V | UCL Centre for Cardiovascular Imaging, Institute of Cardiovascular Science, London, UK |
| Olivieri, LJ | The Heart and Vascular Institute, UPMC Children's Hospital of Pittsburgh, Pittsburgh, PA |
| Quail, M | UCL Centre for Cardiovascular Imaging, Institute of Cardiovascular Science, London, UK |
| Raimondi, F | Congenital Cardiology Unit, Ospedale Papa Giovanni XXIII, Bergamo, Italy |
| Ramachandran, P | Division of Pediatric Cardiology, University of Kentucky, Lexington, KY |
| Rathod, RH | Department of Cardiology, Boston Children’s Hospital, Boston, MA |
| Renella, P | Division of Pediatric Cardiology, CHOC Children’s Hospital, Orange, CA |
| Renno, MS | Division of Pediatric Cardiology, Arkansas Children's Hospital, Little Rock, AR |
| Robinson, JD | Division of Pediatric Cardiology, Ann & Robert H. Lurie Children's Hospital, Chicago, IL |
| Ruchira, G | Division of Pediatric Cardiology, Cedars-Sinai Guerin Children's Hospital, Los Angeles, CA |
| Shah, A | Division of Pediatric Cardiology, New York-Presbyterian Morgan Stanley Children’s Hospital, New York, NY |
| Slesnick, TC | Division of Pediatric Cardiology, Children's Healthcare of Atlanta, Atlanta, GA |
| Soslow, JH | Division of Pediatric Cardiology, Vanderbilt University Medical Center, Nashville, TN |
| Steele, J | Division of Pediatric Cardiology, Yale New Haven Children's Hospital, New Haven, CT |
| Stern, KW | Division of Pediatric Cardiology, Mount Sinai Kravis Children's Hospital, New York, NY |
| Thattaliyath, B | Division of Pediatric Cardiology, Stead Family Children’s Hospital, Iowa City, IA |
| Vaikom House, A | Division of Pediatric Cardiology, Oklahoma Children's Hospital, Oklahoma City, OK |
| Weigand, J | Division of Pediatric Cardiology, Texas Children's Hospital, Houston, TX |
